# Adipose Tissue Insulin Resistance in South Asian and Nordic Women after Gestational Diabetes Mellitus

**DOI:** 10.3390/metabo14050288

**Published:** 2024-05-18

**Authors:** Ahalya Anita Suntharalingam Kvist, Archana Sharma, Christine Sommer, Elisabeth Qvigstad, Hanne Løvdal Gulseth, Stina Therese Sollid, Ingrid Nermoen, Naveed Sattar, Jason Gill, Tone Møller Tannæs, Kåre Inge Birkeland, Sindre Lee-Ødegård

**Affiliations:** 1 Institute of Clinical Medicine, University of Oslo, 0372 Oslo, Norway; a.a.s.kvist@medisin.uio.no (A.A.S.K.); arcshar@gmail.com (A.S.); elisabeth.qvigstad@medisin.uio.no (E.Q.); ingrid.nermoen@medisin.uio.no (I.N.); k.i.birkeland@medisin.uio.no (K.I.B.); 2 Department of Endocrinology, Morbid Obesity and Preventive Medicine, Oslo University Hospital, 0424 Oslo, Norway; christine.sommer@medisin.uio.no; 3 Department of Endocrinology, Akershus University Hospital, 1478 Lørenskog, Norway; 4 Norwegian Institute of Public Health, 0213 Oslo, Norway; hannelovdal.gulseth@fhi.no; 5 Department of Medicine, Drammen Hospital, Vestre Viken Health Trust, 3004 Drammen, Norway; stthso@vestreviken.no; 6 School of Cardiovascular and Metabolic Health, University of Glasgow, BHF Glasgow Cardiovascular Research Centre, 126 University Place, Glasgow G12 8TA, UK; naveed.sattar@glasgow.ac.uk (N.S.); jason.gill@glasgow.ac.uk (J.G.); 7 EpiGen, Medical Division, Akershus University Hospital, 1478 Lørenskog, Norway; t.m.tannas@medisin.uio.no

**Keywords:** South Asian, type 2 diabetes, non-esterified fatty acids, pre-hepatic insulin, insulin clearance, insulin resistance, adipose tissue, fetuin-A, inflammation, liver fat, gestational diabetes

## Abstract

South Asians (SAs) have a higher risk of developing type 2 diabetes (T2D) than white Europeans, especially following gestational diabetes mellitus (GDM). Despite similar blood glucose levels post-GDM, SAs exhibit more insulin resistance (IR) than Nordics, though the underlying mechanisms are unclear. This study aimed to assess markers of adipose tissue (AT) IR and liver fat in SA and Nordic women post-GDM. A total of 179 SA and 108 Nordic women in Norway underwent oral glucose tolerance tests 1–3 years post-GDM. We measured metabolic markers and calculated the AT IR index and non-alcoholic fatty liver disease liver fat (NAFLD-LFS) scores. Results showed that normoglycaemic SAs had less non-esterified fatty acid (NEFA) suppression during the test, resembling prediabetes/T2D responses, and higher levels of plasma fetuin-A, CRP, and IL-6 but lower adiponectin, indicating AT inflammation. Furthermore, normoglycaemic SAs had higher NAFLD-LFS scores, lower insulin clearance, and higher peripheral insulin than Nordics, indicating increased AT IR, inflammation, and liver fat in SAs. Higher liver fat markers significantly contributed to the ethnic disparities in glucose metabolism, suggesting a key area for intervention to reduce T2D risk post-GDM in SAs.

## 1. Introduction

South Asian individuals are at higher risk of type 2 diabetes compared with their counterparts of white European descent [1,2], especially when living in high-income countries, such as Norway [3]. South Asians often develop type 2 diabetes 5–15 years earlier than white Europeans and at a lower body mass index (BMI) [2,4]. Furthermore, type 2 diabetes risk after gestational diabetes mellitus may be higher in South Asians compared to white Europeans [5,6].

We recently reported that, amongst women who remained normoglycaemic after gestational diabetes mellitus, South Asian women had lower hepatic insulin clearance (HIC) and lower insulin secretion relative to insulin resistance compared with their Nordic counterparts [7]. The metabolic phenotype of these normoglycaemic South Asians was comparable to the Nordic women who had progressed to prediabetes/type 2 diabetes [7]. However, the mechanisms behind the higher risk for type 2 diabetes after gestational diabetes mellitus in South Asians remain incompletely understood [1,8].

South Asians tend to have higher insulin resistance, lower beta-cell insulin secretion, and more ectopic fat in the liver than age- and BMI-matched white Europeans [1,2,4,9]. Ectopic lipid deposition is believed to be a major factor in the development of insulin resistance [10,11]. Ectopic lipids in the liver are strongly related to increased non-esterified fatty acids (NEFAs) release from adipose tissue [10,11,12]. Higher levels of NEFAs are seen with increased adipose tissue inflammation and adipocyte insulin resistance, often as a result of energy surplus [13]. NEFA may then accumulate in the liver and initiate non-alcoholic fatty liver disease (NAFLD) [14], and increase concentrations of lipid intermediates that ultimately block the insulin receptor pathway in hepatocytes [11,15]. Hepatocellular insulin resistance enhances gluconeogenesis and raises glucose production beyond the body’s needs [11,15]. In addition, increased pre-hepatic insulin levels in response to insulin resistance may facilitate lipogenesis in the liver and reduced first-pass insulin clearance that may lead to hyperinsulinaemia [16].

Furthermore, increased NEFA availability and ectopic liver fat may upregulate the expression of hepatokines, such as fetuin-A [17]. Fetuin-A may activate adipocyte Toll-like receptor 4 (TLR4) signalling and induce insulin resistance in adipocytes [18,19,20]. Hence, there seems to be inter-tissue communication between the adipose tissue and the liver in insulin resistance [10,11,17]. We have previously shown that fetuin-A may mediate the difference in adipose tissue insulin resistance between South Asians and Nordics with overt type 2 diabetes [21]. However, similar data on women without type 2 diabetes are lacking.

Here, we analysed South Asian and Nordic women up to three years after gestational diabetes mellitus, who had either remained normoglycaemic or had progressed to prediabetes/type 2 diabetes. We assessed ethnic differences in NEFA suppression during an oral glucose tolerance test (OGTT) as a marker of adipose tissue insulin resistance. We also assessed the markers of liver fat content and adipose tissue inflammation and measured plasma fetuin-A levels to obtain indications of adipose tissue and liver crosstalk for diabetes risk in South Asians.

## 2. Materials and Methods

As described in detail previously [3,7], the DIAbetes in South Asians 1 (DIASA 1) study was approved by the South-Eastern Norway Regional Committee for Medical and Health Research Ethics (reference number: 2018/689). All participants gave written informed consent. Briefly, DIASA 1 included women from the Oslo area in Norway. The women had previous gestational diabetes mellitus according to the WHO 1999 [22] or modified International Association of Diabetes and Pregnancy Study Group (IADPSG) criteria [23] one to three years before investigation. Inclusion criteria were adulthood (age equal to or above 18 years) and South Asian or Nordic ethnic origin. Exclusion criteria were new pregnancies after the index pregnancy, exclusive breastfeeding at the time of examination, known diabetes before the index pregnancy or at the time of examination, ongoing inflammatory or serious disease, or a history of major surgical procedures less than three months before inclusion. DIASA 1 included in total 287 (110 Pakistani, 33 Indian, 5 Bangladeshi, 31 Sri Lankan, 101 Norwegian, 3 Swedish, 3 Danish, and 1 Icelandic) women.

At the study visit, all women underwent a 5-point OGTT with 75 g of anhydrous glucose in the morning after at least eight hours of fasting. Blood was collected at 0, 15, 30, 60, and 120 min during the OGTT in cooled sodium fluoride tubes for glucose analysis and kept on ice until centrifugation at 4 degrees Celsius, and serum-separating tubes were used for analyses of insulin and C-peptide, and centrifuged after 30 min. Serum and plasma were separated for immediate analyses or frozen at minus 80 degrees Celsius for later analyses. We also measured height, weight, waist, and hip circumferences, while clinical data were retrieved from medical records.

Plasma glucose was analysed by enzymatic photometry (Roche Diagnostics, Mannheim, Germany), and serum insulin was analysed by electrochemiluminescence immunoassay (Cobas e601, Roche Diagnostics). The coefficients of variation (CoV) were 2.5% and 7%, for glucose and insulin, respectively. Plasma NEFA was analysed by an in-house enzymatic colourimetric method, and the CoV was 5.0%. One woman did not donate blood samples for NEFA analysis. Serum leptin levels were measured using an enzyme-linked immunosorbent assay (ELISA) kit (Mediagnost) with a CoV of 10%. Serum adiponectin levels were measured using a competitive radioimmunoassay kit (Merck Millipore, Burlington, MA, USA) and had a CoV of 6.6%. Plasma high-sensitive C-reactive protein (hsCRP) levels were measured using an in-house particle-enhanced immunoturbidimetry method with a CoV of 6%. Plasma interleukin-6 (IL-6) levels were measured using the Elecsys IL-6 kit (electrochemiluminescence immunoassay (ECLIA)) from Roche Diagnostics, and the CoV was 5%. Plasma fetuin-A levels were measured using Human Fetuin A Quantikine ELISA Kit (R&D Systems). The CoV was 7.4%.

The NAFLD liver fat score (NAFLD-LFS) was calculated by the following formula: −2.89 + 1.18 × (metabolic syndrome [24]: yes = 1, no = 0) + 0.45 × (type 2 diabetes: yes = 2, no = 0) + 0.15 × (fasting serum insulin, mU/L) + 0.04 × (ASAT, IU/L) − 0.94 × (ASAT/ALT). We chose the NAFLD-LFS as our main index of liver fat because of its superior prediction of 1H-MRS measured liver fat content and validation in low-risk populations for NAFLD [24]. Furthermore, NAFLD-LFS has also been validated in some Asian populations (Koreans [24] and Indian Asians [25]). However, NAFLD-LFS correlated with high significance with other liver fat indexes (Supplementary Table S1). The AT-IR index was calculated as fasting insulin (pmol/L) x NEFA concentrations (mmol/L) [20,21]. A high index score indicates adipose tissue insulin resistance.

Prediabetes was defined according to the WHO International Expert Committee criteria; fasting plasma glucose 6.1–6.9 mmol/L or 2 h plasma glucose 7.8–11.0 mmol/L or HbA_1c_ 42–47 mmol/mol (6.0–6.4%) [22,26]. Diabetes was defined according to the internationally agreed criteria (HbA_1c_ ≥ 48 mmol/mol and/or fasting plasma glucose ≥ 7.0 mmol/L and/or 2 h plasma glucose ≥ 11.1 mmol/L), but a diagnostic value was not confirmed by a second test [27,28].

Group comparisons were performed using Wilcoxon ranked tests. Differences in time responses to the OGTT between South Asians and Nordics were modelled using random intercept mixed models from the lme4 R package. The total area under the curve (AUC) was calculated using the trapezoid rule. Bivariate correlations were performed using Spearman’s ranked test. OGTT data are presented as medians and interquartile ranges (IQR), group comparisons are presented as box plots, and bivariate correlations are presented as scatter plots. We corrected for multiple testing using the Benjamini–Hochberg method, accepting a false discovery rate of 10% (a maximum of 1 out of 10 false positive results). We used multiple regression to test if a covariable (e.g., plasma fetuin-A levels) affected the association between ethnicity and a particular outcome (e.g., the AT-IR index). The *p*-value for the explained effect was estimated using the mediation R package with 1000 bootstraps. We used R version 4.1.3.

## 3. Results

At a median (IQR) of 16.5 (12.1) months after delivery, 31% of South Asian (total *n* = 178) and 53% of Nordic (total *n* = 108) women had a normal OGTT (Table 1 and Supplementary Table S2). South Asian women had higher parity, more first-degree family members with diabetes, and fewer years of education than Nordic women (Table 1). Body mass index (BMI) did not differ between the groups, but normoglycaemic South Asian women had higher waist–height ratios (WHtR). South Asian women were slightly younger than Nordic women (Table 1).

### 3.1. Markers of Adipose Tissue Insulin Resistance

**Figure 1:**
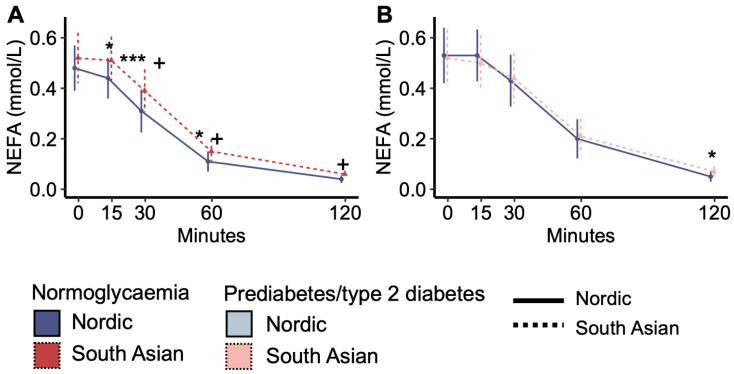
Plasma non-esterified fatty acid (NEFA) concentrations during the oral glucose tolerance test. (**A**) Plasma NEFA concentrations in normoglycaemic women and (**B**) in women with prediabetes/type 2 diabetes. + *p* < 0.05 interaction effect between the ethnic groups from time 0. * *p* < 0.05, and *** *p* < 0.001 ethnic group difference. Data are medians with interquartile ranges. NO = Nordic. SA = South Asian. Blue colour and solid line = Nordics with normoglycaemia. Red colour with stapled line = South Asians with normoglycaemia. Light blue colour with solid line = Nordics with prediabetes/type 2 diabetes. The light red colour and stapled line = South Asians with prediabetes/type 2 diabetes.

Fasting plasma NEFA concentrations were similar between South Asian and Nordic women with normoglycaemia, but the decrease in NEFA concentrations in response to the OGTT was steeper for Nordics than South Asians, and NEFA concentrations were subsequently higher in South Asians than Nordics at 15, 30, and 60 min (Figure 1A). In comparison, plasma NEFA concentrations during the OGTT were similar between South Asian and Nordic women with prediabetes/type 2 diabetes, except for slightly higher levels in South Asians than in Nordics at 120 min (Figure 1B). The response pattern in normoglycaemic South Asians was similar to the response pattern in women with prediabetes/type 2 diabetes (Figure 1A vs. Figure 1B).

**Figure 2:**
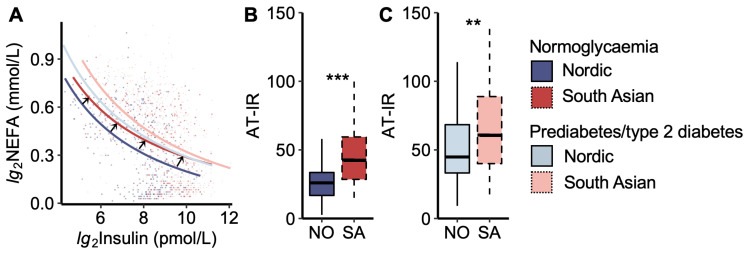
Adipose tissue insulin resistance index. (**A**) Plasma non-esterified fatty acid (NEFA) vs. insulin concentrations during the oral glucose tolerance test. The black arrows indicate that the curve for normoglycaemic South Asians is shifted upwards to the right compared to normoglycaemic Nordics, approaching the curves for the prediabetes/type 2 diabetes groups. The coloured dots represent each women. (**B**) The adipose tissue insulin resistance index (AT-IR), the product of fasting NEFA and insulin levels, in normoglycaemic women and (**C**) women with prediabetes/type 2 diabetes. NO = Nordic. SA = South Asian. Box plots show medians, 25–75 percentiles, and min/max ranges. ** *p* < 0.01, and *** *p* < 0.001 ethnic group difference.

We then analysed the adipose tissue insulin resistance curve, which describes the constant product of peripheral plasma insulin and NEFA levels during the OGTT (Figure 2). We noticed that the curve for South Asians with normoglycaemia was moved up and to the right compared to that of Nordics with normoglycaemia, thus approaching the curves for women with prediabetes/type 2 diabetes (Figure 2A).

Another way of visualizing the relationship between plasma NEFA and insulin levels is by calculating the AT-IR index (product of insulin and NEFA levels). The AT-IR index was higher in South Asians than in Nordics, both in the normoglycaemic and prediabetes/type 2 diabetes groups (Figure 2B,C). No significant difference was observed between normoglycaemic South Asians and Nordics with prediabetes/type 2 diabetes.

### 3.2. Markers of Adipose Tissue Inflammation and Fat Mass

Plasma concentrations of hsCRP, IL-6, and leptin were higher in South Asians than in Nordics with normoglycaemia, but similar between the ethnicities in the prediabetes/type 2 diabetes group (Supplementary Table S3). Plasma adiponectin levels were lower in South Asians than in Nordics in both the normoglycaemic and prediabetes/type 2 diabetes groups (Supplementary Table S3). Spearman’s correlations implied that the AT-IR index was positively associated with plasma levels of hsCRP, IL-6, and leptin and negatively associated with plasma adiponectin levels (Supplementary Table S4).

### 3.3. Markers of Liver Fat and Insulin Resistance and Associations with Post-Prandial Glucose Levels

**Figure 3:**
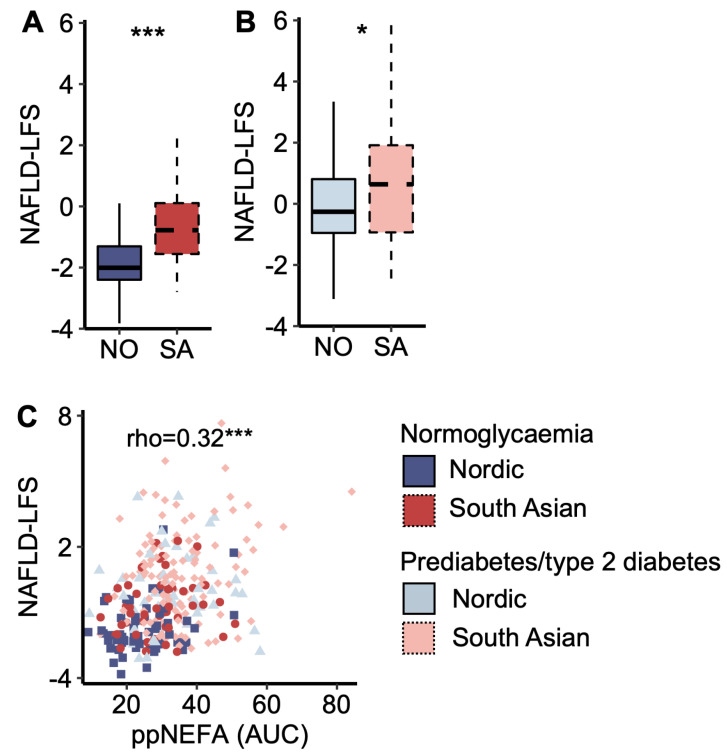
A marker of liver fat content. (**A**) The non-alcoholic fatty liver disease liver fat score (NAFLD-LFS) in Nordic and South Asian women with normoglycaemia and (**B**) prediabetes/type 2 diabetes. (**C**) A scatter plot of NAFLD-LFS scores and post-prandial (pp) non-esterified fatty acid (NEFA) concentration (area under the curve (AUC) from the oral glucose tolerance test (OGTT)). * *p* < 0.05 and *** *p* < 0.001. Box plots show medians, 25–75 percentiles, and min/max ranges. NO = Nordic. SA = South Asian.

South Asians displayed a higher NAFLD-LFS score than Nordics, both in the normoglycaemic and prediabetes/type 2 diabetes groups (Figure 3 A,B). The NAFLD-LFS score correlated to post-prandial (AUC) plasma NEFA concentrations (Figure 3C). Normoglycaemic South Asians displayed higher post-prandial (AUC OGTT) glucose levels than normoglycaemic Nordics (7%, *p* = 0.008), and the NAFLD-LFS score explained 74.1% of the ethnic difference in post-prandial (AUC OGTT) plasma glucose concentrations (Supplementary Figure S1). Similar results were obtained when running the same analysis in the normoglycaemic and prediabetes/type 2 diabetes groups separately. The explanatory effect of the NAFLD-LFS score on glucose levels was not present when substituting post-prandial with fasting glucose (*p* = 0.09) but persisted when substituting with 2 h glucose levels (*p* = 0.004).

### 3.4. Fetuin-A and Adipose Tissue Insulin Resistance

**Figure 4:**
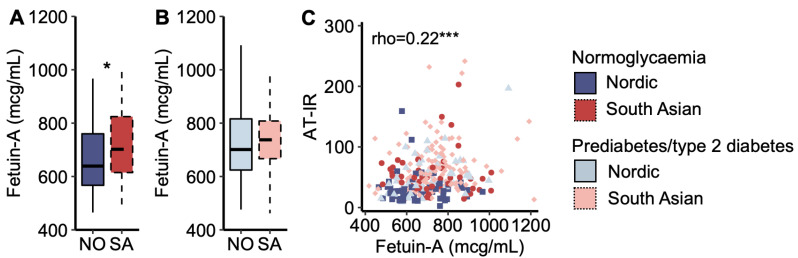
Fetuin-A. (**A**) Plasma fetuin-A concentrations in Nordic and South Asian women with normoglycaemia and (**B**) prediabetes/type 2 diabetes. (**C**) A scatter plot of adipose tissue insulin resistance (AT-IR) indexes as a function of plasma fetuin-A concentrations. * *p* < 0.05, and *** *p* < 0.001. Box plots show medians, 25–75 percentiles, and min/max ranges. NO = Nordic. SA = South Asian.

Plasma fetuin-A concentration was higher in South Asians with normoglycaemia than in Nordics with normoglycaemia (Figure 4A), but it was similar between the ethnicities for women with prediabetes/type 2 diabetes (Figure 4B). Plasma fetuin-A concentrations correlated positively with the AT-IR index across all women (Figure 4C). In multiple regression analysis, plasma fetuin-A levels explained 10.0% of the ethnic difference in the AT-IR index.

As demonstrated in several recent publications [18,19,20], we also observed that the association between plasma fetuin-A levels and the AT-IR index was stronger with increasing fasting plasma NEFA concentrations [standardized beta (B_std_) = 0.11 and *p* = 0.034 increase in the AT-IR index per standard deviation increase in plasma fetuin-A levels interacted with fasting NEFA levels (mcg/mL × mmol/L)]. The results were B_std_ = 0.10 and *p* = 0.045 when substituting fasting for post-prandial (AUC) NEFA levels.

## 4. Discussion

The main results in this study were that despite normoglycaemia, South Asian women displayed higher adipose tissue insulin resistance than Nordic women when examined 1–3 years after gestational diabetes mellitus. Pregnancy can be regarded as a ‘metabolic stress test’ [29], and alterations related to gestational diabetes mellitus are strongly associated with future risk of type 2 diabetes, hypertension, hyperlipidaemia, and cardiovascular disorders [29]. Based on our data, this risk seems stronger for South Asian than Nordic women.

Increased adipose tissue insulin resistance, as captured by the AT-IR index, and increased plasma NEFA levels were associated with a marker of higher liver fat content, plasma fetuin-A levels, and impaired post-prandial glucose response in South Asians. Adipose tissue excess and dysfunction are believed to be major pathogenic factors in the development of type 2 diabetes [10], in particular in people of South Asian origin [21]. Hence, it is interesting that normoglycaemic South Asians displayed clear signs of adipose tissue insulin resistance, as indicated by the impaired suppression of NEFA levels during the OGTT. Both plasma NEFAs themselves, but perhaps more importantly, plasma NEFA levels relative to plasma insulin levels are clear indicators of adipose tissue insulin resistance [30]. We observed that, in normoglycemic South Asian women, the relationship curve between plasma NEFA and insulin levels was more similar to that seen in women with prediabetes/type 2 diabetes. This further suggests that South Asian normoglycemic women experience adipose tissue insulin resistance.

Several studies now indicate that adipose tissue insulin resistance may be a consequence of adipose tissue hypertrophy/hyperplasia and inflammation [10,11,31]. Although we did not measure these factors directly, we observed increased plasma IL-6, hsCRP, and leptin levels and decreased plasma adiponectin levels in normoglycaemic South Asians. Plasma IL-6, hsCRP, and leptin were positively correlated, whereas plasma adiponectin levels were negatively correlated to markers of adipose tissue insulin resistance. These results are in line with previous studies [32,33,34]. Hence, our data imply that adipose tissue insulin resistance in normoglycaemic South Asians may be related to adipose tissue hypertrophy/hyperplasia and inflammation [10,11,31].

Adipose tissue insulin resistance, and thus increased lipolysis instead of lipid storage in the post-prandial state, is a major contributor to ectopic lipid deposition, especially in the liver [10,11,35]. Although we did not direct quantify liver fat content, we calculated the NAFLD-LFS index for liver fat. We chose NAFLD-LFS because it was designed based on 1H magnetic resonance imaging [36] and validated in low-risk populations for NAFLD [24] and some Asian populations (Koreans [24] and Indian Asians [25]). However, NAFLD-LFS was strongly correlated to other indexes of liver fat content. As expected, we observed high NAFLD-LFS scores in normoglycaemic South Asians and clear associations between NAFLD-LFS and plasma NEFA levels. Furthermore, NAFLD-LFS explained most of the ethnic differences in post-prandial glucose levels in our data. This is interesting because increased liver fat may lead to the accumulation of lipid intermediates, such as *sn*1,2-diacylglycerol, that block insulin signalling and increase gluconeogenesis [10,37]. Hence, our data indicate that insulin resistance related to pregnancy may influence metabolic disturbances other than just glucose levels, such as lipid metabolism [38]. A post-partum follow-up of, e.g., triglycerides levels [29] could help to identify women at increased risk for type 2 diabetes and related complications.

Although our data indicate that a surplus of NEFAs from adipose tissue may affect the liver, our data also indicate that liver metabolism could influence adipose tissue insulin resistance through hepatokine secretion [17]. Plasma levels of the hepatokine fetuin-A were elevated in normoglycaemic South Asians and explained 10% of the ethnic difference in adipose tissue insulin resistance. The relationship between fetuin-A and adipose tissue insulin resistance was also stronger at higher NEFA levels. These observations match several reports that demonstrated an interaction between fetuin-A and fatty acids to induce adipose tissue insulin resistance [18,19,20]. A growing body of evidence is now linking fetuin-A to adipose tissue insulin resistance by inducing TLR4 signalling [17,18,19,20,21], which might seem especially important in South Asians [21]. Our data are also in line with indications of a ‘vicious cycle’ between enhanced lipolysis, increased liver fat content, and NEFA-induced fetuin-A expression [39] that in turn worsens adipose tissue insulin resistance and lipolysis.

Strengths of our study include a unique study population, a decent sample size, and several layers of data to indicate adipose tissue and liver crosstalk in insulin resistance. Although we have women in a range from normoglycaemia to prediabetes/type 2 diabetes, the cross-sectional design is a limitation, and our data cannot imply causality. The women were recruited from outpatient clinics and thus represent a high-risk population, which might limit generalizability. Also, most of our data are based on indirect measures, such as indexes for insulin resistance and liver fat content. The preferred methods would have been the hyperinsulinaemic euglycaemic clamp to quantify insulin resistance, and 1H magnetic resonance imaging to measure liver fat content. Harvesting adipose tissue and skeletal muscle biopsies would have provided important tissue-specific data. Future studies should also aim to include Nordic and South Asian women without previous gestational diabetes mellitus. Furthermore, no data on exercise and diet were collected for the groups in this study, and differences in diet and exercise could potentially explain some of the observed ethnic differences in metabolic outcomes.

## 5. Conclusions

South Asian women with normoglycaemia investigated 1–3 years after gestational diabetes mellitus displayed increased adipose tissue insulin resistance and elevated markers of liver fat content, as compared to Nordic women with normoglycaemia. The normoglycaemic South Asian women displayed similar metabolic profiles as women with prediabetes/type 2 diabetes. These novel observations may partly explain the increased risk for type 2 diabetes after gestational diabetes mellitus in South Asian women. However, more research is needed to examine adipose tissue function and morphology in South Asians vs. Nordics and their change with weight gain.

## Figures and Tables

**Table 1:** Subject characteristics.

	Normoglycaemia		*p*	Prediabetes/Type 2 Diabetes		*p*
	Nordics	South Asians		Nordics	South Asians	
Participants, *n* (%)	57/108 [52.7]	55/178 [30.9]		51/108 [47.2]	123/178 [69.1]	<0.001 *
Age (years)	36.7 (4.9)	34.4 (3.9)	<0.001	36.2 (4.8)	34.6 (4.2)	0.031
Years since index pregnancy	1.4 [0.9]	1.3 [1.2]	0.093	1.6 [1.1]	1.3 [1.0]	0.159
HbA_1c_ (mmol/mol)	35.0 (0.3)	36.6 (0.5)	<0.001	38.0 (0.6)	40.4 (0.4)	0.004
HbA_1c_ (%)	5.4 (2.1)	5.5 (2.2)		5.6 (2.2)	5.8 (2.2)	
Weight [kg]	72.0 [24.0]	68.8 [17.6]	<0.001	86.0 [21.0]	72.9 [19.3]	<0.001
Height (cm)	167.9 (6.0)	158.1(6.0)	<0.001	165.8 (6.0)	160.0 (6.6)	<0.001
BMI [kg/m^2^]	25.5 [8.4]	27.6 [5.4]	0.663	31.9 [6.9]	28.8 [6.4]	0.007
Waist circumference (cm)	92.2 (14.3)	93.6 (12.6)	0.777	100.5 (12.4)	97.9 (11.5)	0.185
Waist–hip ratio	0.87 (0.09)	0.88 (0.06)	0.018	0.89 (0.08)	0.91 (0.07)	0.069
Waist–height ratio	0.55 (0.86)	0.59 (0.77)	0.002	0.61 (0.08)	0.61 (0.07)	0.657
Total cholesterol (mmol/L)	4.24 (0.82)	4.2 (0.6)	0.358	4.3 (0.8)	4.4 (0.7)	0.397
ASAT (U/L)	20.7 (4.3)	24.3 (5.9)	0.004	23.8 (7.7)	24.2 (5.2)	0.685
ALAT (U/L)	16.3 (6.1)	20.4 (9.1)	0.065	22.8 (13.9)	22.1 (8.4)	0.668
Parity (no.)	1.7 (0.7)	2.1 (1.0)	<0.001	1.7 (0.7)	2.2 (1.0)	0.001
Prior GDM (yes/no [%])	9/57 [16]	13/54 [24]	0.100	15/51 [29]	42/121 [35]	0.503
Family history of diabetes (yes/no [%])	10/46 [22]	38/51 [75]	<0.001	12/45 [27]	87/117 [74]	<0.001
Insulin use in pregnancy (yes/no [%])	11/57 [19]	19/55 [35]	0.138	23/51 [45]	53/123 [43]	0.809
Years of education	17.2 (2.8)	14.4 (3.3)	<0.001	16.3 (3.1)	15.0 (3.5)	0.022
Smoking			<0.001			<0.001
Daily (yes/no [%])	1/57 (1.8)	2/55 (3.6)		7/51 (13.7)	2/123 (1.6)	
Earlier (yes/no [%])	18/57 (31.6)	2/55 (3.6)		20/51 (39.2)	2/123 (1.6)	
Never (yes/no [%])	38/57 (66.7)	51/55 (92.7)		24/51 (47.1)	119/123 (96.8)	
Alcohol consumption			<0.001			<0.001
Weekly (yes/no [%])	18/57 (31.6)	1/55 (1.8)		10/51 (19.6)	2/123 (1.6)	
Less than weekly (yes/no [%])	38/57 (66.7)	8/55 (14.6)		38/51 (74.5)	12/123 (9.8)	
Never (yes/no [%])	1/57 (1.8)	46/55 (83.6)		3/51 (5.9)	109/123 (88.6)	

Data are mean (standard deviation) or median [interquartile range] or numbers [per cent, %], as appropriate. No. = numbers. Family history = first-degree relative with diabetes. GDM = gestational diabetes before index pregnancy. ASAT = aspartate transaminase. ALAT = alanine transaminase. *p* = *p*-value from a Wilcoxon’s rank test or a Student *t*-test, as appropriate. * Fisher’s exact test for numbers.

## Data Availability

The original contributions presented in the study are included in the article/supplementary material, and further inquiries can be directed to the corresponding author.

## Supplementary Materials

The following supporting information can be downloaded at: https://www.mdpi.com/article/10.3390/metabo14050288/s1; Supplementary Figure S1, Supplementary Tables S1–S4. Supplementary Figure S1. Marker of liver fat content and influence on post-prandial glucose levels. Supplementary Table S1. Correlations between the NAFLD liver fat score (NAFLD-LFS) and other liver fat indexes. Supplementary Table S2. Plasma glucose and insulin responses to the 2-hour oral glucose tolerance test. Supplementary Table S3. Ethnic differences in cytokine levels. Supplementary Table S4. Correlations between AT-IR and cytokines.
