# Supplementary material for: Adipose Tissue Insulin Resistance in South Asian and Nordic Women after Gestational Diabetes Mellitus
Source: Metabolites. 2024 May 18;14(5):288. doi: 10.3390/metabo14050288 (PMC11123011; doi:10.3390/metabo14050288)
Supplement: Supplementary file 1 [file metabolites-14-00288-s001.zip › DIASA_NEFA_290324_Supplementary_Figures.pdf]

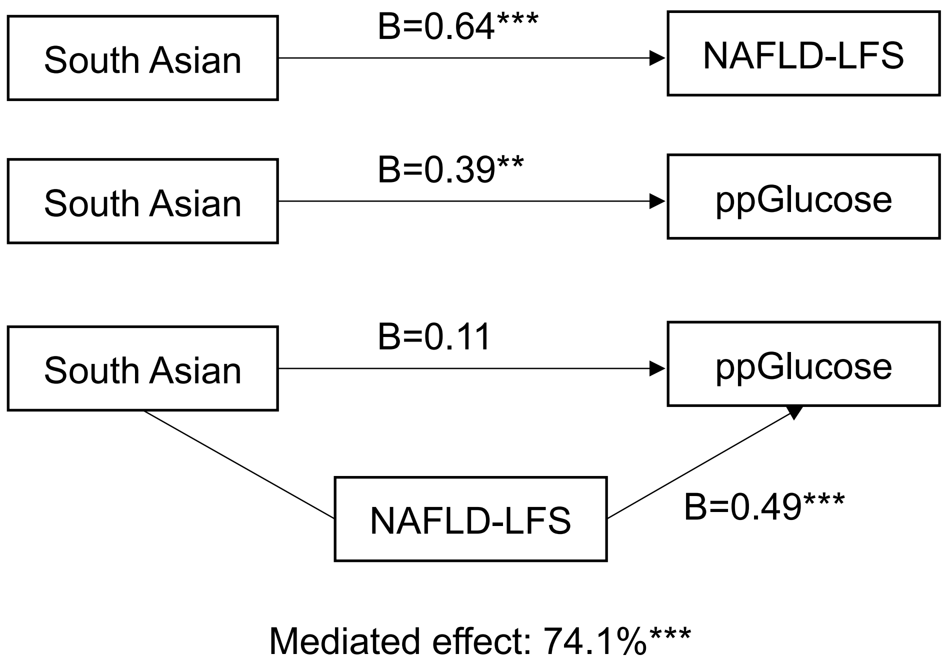

Supplementary Figure 1. Marker of liver fat content and influence on post-prandial glucose levels. The non-alcoholic fatty liver disease liver fat score (NAFLD-LFS) and post-prandial (pp) (areal under the curve = AUC from the oral glucose tolerance test = OGTT) glucose were higher in Nordic than south Asian women. The NAFLD-LFS score explained most of the ethnic difference in pp glucose levels. *p<0.05, **p<0.01 and ***p<0.001.
