# Supplementary material for: Adipose Tissue Insulin Resistance in South Asian and Nordic Women after Gestational Diabetes Mellitus
Source: Metabolites. 2024 May 18;14(5):288. doi: 10.3390/metabo14050288 (PMC11123011; doi:10.3390/metabo14050288)
Supplement: Supplementary file 1 [file metabolites-14-00288-s001.zip › DIASA_NEFA_290324_Supplementary_Tables.pdf]

Supplementary Table 1. Correlations between the NAFLD liver fat score (NAFLD-LFS) and other liver fat indexes.

Index	Rho	p
FSI	0,64	6,29 x 10^{-32}
HSI	0,62	3,16 x 10^{-29}
ZJU	0,60	7,74 x 10^{-28}

Hepatic steatosis index (HSI) = 8 × ALT/AST ratio + BMI (+2 if type 2 diabetic; +2 if female).

ZJU index = BMI (kg/m^2) + fasting plasma glucose (mmol/L) + TG (mmol/L) + 3 × ALT/AST ratio (+2 if female).

NAFLD liver fat score (NAFLD-LFS) = −2.89 + 1.18 × (metabolic syndrome—yes = 1, no = 0) + 0.45 × (type 2 diabetes—yes = 2, no = 0) + 0.15 × (fasting serum insulin, mU/L) + 0.04 × (AST, IU/L) − 0.94 × (AST/ALT).

Framingham steatosis index (FSI) = $e^x/(1 + e^x)$, where X = −7.981 + 0.011 × age (years) − 0.146 × sex (female = 1, male = 0) + 0.173 × BMI (kg/m^2) + 0.007 × TG (mg/dL) + 0.593 × hypertension (yes = 1, no = 0) + 0.789 × diabetes (yes = 1, no = 0) + 1.1 × ALT:AST ratio ≥ 1.33 (yes = 1, no = 0).

HSI: 10.1016/j.dld.2009.08.002
ZJU: 10.1109/ULTSYM.2015.0283
FSI: 10.1016/j.cgh.2016.03.034
NAFLD-LFS: 10.1053/j.gastro.2009.06.005

Supplementary Table 2. Plasma glucose and insulin responses to the 2-hour oral glucose tolerance test

	Normoglycaemia			Prediabetes/type 2 diabetes		
	Nordics	South Asians	*P*	Nordics	South Asians	*P*
Glucose (mmol/L)						
Time 0	5.4 [0.6]	5.5 [0.6]	0.189	6.0 [1.0]	5.9 [1.0]	0.680
Time 15	6.3 [1.1]	6.7 [0.9]	0.101	7.2 [1.6]	7.3 [1.6]	0.813
Time 30	7.9 [1.4]	8.4 [1.4]	0.005	9.2 [2.4]	9.3 [2.4]	0.987
Time 60	7.6 [2.0]	8.5 [2.4]	0.016	10.8 [2.3]	11.1 [2.3]	0.509
Time 120	6.1 [1.7]	6.8 [1.2]	0.076	9.0 [2.7]	9.4 [2.7]	0.826

Data are median and [interquartile range]. P = p-value from a Wilcoxon's rank test.

Supplementary Table 3. Ethnic differences in cytokine levels

	Normoglycaemia			Prediabetes/type 2 diabetes		
	Nordics	South Asians	*P*	Nordics	South Asians	*P*
Leptin	1036 [826]	1620 [1028]	0.001	1824 [1839]	1659 [1405]	0.868
Adiponectin	10.9 [5.2]	7.4 [3.6]	1.6×10^{-6}	8.7 [7.1]	6.8 [3.8]	0.002
IL-6	1.5 [0.4]	2.2 [1.5]	0.0002	1.9 [1.4]	2.3 [1.9]	0.091
hsCRP	0.8 [1.9]	1.7 [2.3]	0.008	2.2 [4.8]	2.7 [3.3]	0.811

Data are median and [interquartile range]. P = p-value from a Wilcoxon's rank test.

Supplementary Table 4. Correlations between AT-IR and cytokines

	Rho	P-value
Leptin	0.50	$<2.2x10^{-16}$
Adiponectin	-0.41	$<3.0x10^{-11}$
IL-6	0.48	$<2.2x10^{-16}$
hsCRP	0.42	$<7.3x10^{-13}$

Spearman's correlations
